# Induction of Histiocytic Sarcoma in Mouse Skeletal Muscle

**DOI:** 10.1371/journal.pone.0044044

**Published:** 2012-08-31

**Authors:** Jianing Liu, Simone Hettmer, Michael D. Milsom, Inga Hofmann, Frederic Hua, Christine Miller, Roderick T. Bronson, Amy J. Wagers

**Affiliations:** 1 Howard Hughes Medical Institute, Department of Stem Cell and Regenerative Biology, Harvard University, Harvard Stem Cell Institute, and Joslin Diabetes Center, Cambridge, Massachusetts, United States of America; 2 Department of Pediatric Oncology, Dana Farber Cancer Institute and Division of Pediatric Hematology/Oncology, Children's Hospital, Boston, Massachusetts, United States of America; 3 HI-STEM (Heidelberg Institute for Stem Cell Technology and Experimental Medicine) and DKFZ (German Cancer Research Center), Heidelberg, Germany; 4 Department of Biomedical Sciences, Cumming School of Veterinary Medicine at Tufts University Veterinary School, North Grafton, Massachusetts, United States of America; University of Minnesota Medical School, United States of America

## Abstract

Myeloid sarcomas are extramedullary accumulations of immature myeloid cells that may present with or without evidence of pathologic involvement of the bone marrow or peripheral blood, and often coincide with or precede a diagnosis of acute myeloid leukemia (AML). A dearth of experimental models has hampered the study of myeloid sarcomas and led us to establish a new system in which tumor induction can be evaluated in an easily accessible non-hematopoietic tissue compartment. Using ex-vivo transduction of oncogenic *Kras(G12V)* into *p16/p19^−/−^* bone marrow cells, we generated transplantable leukemia-initiating cells that rapidly induced tumor formation in the skeletal muscle of immunocompromised NOD.SCID mice. In this model, murine histiocytic sarcomas, equivalent to human myeloid sarcomas, emerged at the injection site 30–50 days after cell implantation and consisted of tightly packed monotypic cells that were CD48+, CD47+ and Mac1+, with low or absent expression of other hematopoietic lineage markers. Tumor cells also infiltrated the bone marrow, spleen and other non-hematopoietic organs of tumor-bearing animals, leading to systemic illness (leukemia) within two weeks of tumor detection. *P16/p19^−/−^; Kras(G12V)* myeloid sarcomas were multi-clonal, with dominant clones selected during secondary transplantation. The systemic leukemic phenotypes exhibited by histiocytic sarcoma-bearing mice were nearly identical to those of animals in which leukemia was introduced by intravenous transplantation of the same donor cells. Moreover, murine histiocytic sarcoma could be similarly induced by intramuscular injection of *MLL-AF9* leukemia cells. This study establishes a novel, transplantable model of murine histiocytic/myeloid sarcoma that recapitulates the natural progression of these malignancies to systemic disease and indicates a cell autonomous leukemogenic mechanism.

## Introduction

Myeloid sarcomas (also known as chloromas) are extramedullary tumors composed of myeloid lineage cells. Myeloid sarcomas typically present in the setting of acute myeloid leukemia (AML) or in conjunction with transformation of a myelodysplastic syndrome (MDS) [1]. Myeloid sarcomas without bone marrow or peripheral blood involvement often precede the development of new or recurrent leukemia [2]–[4]. Myeloid sarcomas arise predominantly in the bone, soft tissue, lymph nodes, and skin, but essentially any part of the body can be affected [5]–[8]. Treatment of these malignancies generally follows the same therapeutic algorithms established for their systemic, leukemic counterparts and may additionally involve local radiation [2]. The prognostic significance of myeloid sarcoma at first diagnosis of AML remains somewhat unclear. An association with less favorable disease outcomes has been discussed [9], [10], and a recent paper showed that orbital and CNS (central nervous system) myeloid sarcoma in children have a significantly better survival than myeloid sarcoma at other organ sites or AML without myeloid sarcoma [11].

The relative dearth of knowledge regarding the biology of myeloid malignancies arising in extramedullary tissues led us to comparatively evaluate myeloid tumors initiated in either skeletal muscle or in blood following introduction of identical oncogenetic lesions (i.e. oncogenic *KrasG12(V)* and loss of *p16^Ink4A^/p19^Arf^*). Both oncogenic lesions are strongly associated with human and mouse hematopoietic malignancies [12]–[16]. Moreover, constitutively activated *Kras(G12D)* combined with *p16/p19* deficiency induces aggressive cancers in a number of non-hematopoietic tissues and organs in mice [17]–[24]. We therefore introduced oncogenic *Kras* into *p16p19^−/−^* bone marrow cells by ex vivo gene transduction, and then transplanted these genetically altered cells to induce systemic leukemias (by retro-orbital injection), as well as to produce the first transplantable model of murine histiocytic sarcoma (by injection into the gastrocnemius muscles of NOD.SCID mice). Irrespective of transplantation location, tumor cells shared similar morphological and phenotypic features, and histiocytic sarcomas initiated in mouse skeletal muscle seeded systemic disease within weeks of emergence, recapitulating the leukemic progression seen in humans. Finally, murine histiocytic sarcomas could be induced using genetic lesions distinct from *p16/p19^−/−^; Kras*, i.e. *MLL-AF9*, thereby suggesting that the murine histiocytic induction model described here can be applied to study a broad spectrum of hematopoietic malignancies. In summary, this work suggests that the phenotype of hematopoietic neoplasms is largely independent of the tissue environment in which they develop and provides a rapid and reproducible platform to generate and study the behavior of extramedullary hematopoietic tumors.

## Results

### Loss of p16^Ink4A^/p19^Arf^ cooperates with oncogenic Kras(G12V) to induce leukemia

**Figure 1:**
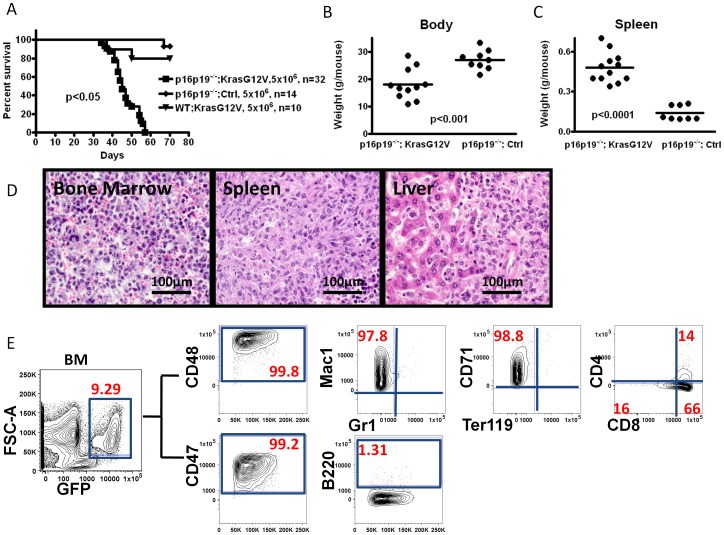
Deficiency in p16^Ink4a^ p19^Arf^ cooperates with oncogenic Kras^G12V^ to produce myeloid leukemia in NOD.SCID mice. (A) Kaplan-Meier survival curve for NOD.SCID recipients after transplantation with C57BL/6 bone marrow (BM) cells modified by the indicated oncogenetic lesions. (B, C) Body weight and spleen weight analyses for NOD.SCID mice receiving *p16p19^−/−^; Kras(G12V)* BM cells via intra-venous transplantation at the time of sacrifice. Kras(G12V) and control groups were injected with cells and sacrificed for analysis at the same time points. (D) Hematoxylin & eosin stain of bone, spleen and liver from NOD.SCID leukemic mice, indicating metastasis of myeloid leukemia cells (60x). (E) Representative flow cytometry analysis of bone marrow from a NOD.SCID recipient of *p16p19^−/−^; Kras(G12V)-GFP* expressing cells. Data are shown as 2-parameter contour plots for Forward Scatter (FSC) or for the indicated cell surface makers. Plots at right show data for cells previously gated for viability (propidium iodide-, not shown) and GFP expression (leftmost plot).

Bone marrow (BM) cells were isolated from *p16p19^−/−^* mice, infected with *Kras(G12V)* in a GFP-tagged pGIPZ lentivirus, and injected retro-orbitally into immunodeficient NOD.SCID mice. All recipient mice showed significant weight loss, anemia and splenomegaly, and were moribund 35–60 days post injection (32 mice evaluated in 4 independent experiments, Fig. 1A–C). In contrast, *p16p19^−/−^* BM cells infected with control (Ctrl) virus (GFP-tagged empty pGIPZ vector) failed to induce leukemia in 12 out of 14 recipients (2 of the 14 recipients died without clinically apparent tumors but could not be subjected to necropsy due to autolysis) (Fig. 1A). Likewise, wild-type (WT) C57BL/6 BM cells infected with *Kras(G12V)* induced leukemias in only 2 out of 10 injected NOD.SCID mice. Thus, consistent with previous reports [15], [25]–[28], the combination of oncogenic *Kras* and *p16p19*-deficiency potently drives leukemogenesis in mouse BM cells, whereas either of these two lesions alone shows limited leukemogenic potential within an 8 to 10- week follow up time. Significantly, prior reports indicate that even with longer follow up (8–9 months), *p16p19*-deficiency or oncogenic *Kras* alone produces hematopoietic neoplasms (mostly B- or T-lymphomas and T cell leukemias) with relatively low efficiency [15], [29].

The spleen and liver of leukemic mice originally injected with *p16p19^−/−^; Kras(G12V)* BM cells, exhibited extramedullary hematopoiesis and massive infiltration by intermediate to large size cells with oval, irregularly folded nuclei, prominent nucleoli, and a moderate to large amount of eosinophilic cytoplasm most consistent with involvement of a non-lymphoid hematopoietic malignancy (Fig. 1D). In contrast, the bone marrow of these mice contained variable foci of immature intermediate to large size cells with round or oval, irregular nuclei, prominent nucleoli and moderate cytoplasm. Peripheral blood smears revealed polychromasia and marked reticulocytosis, suggestive of extramedullary hematopoiesis; however, no blasts were noted in the peripheral blood (data not shown). Taken together, this constellation of findings is consistent with the development of murine histiocytic leukemia, equivalent to human acute myeloid leukemia, after retro-orbital injection of *p16p19^−^/^−^; Kras* bone marrow cells [30].

The immunophenotype of tumor-derived GFP+ cells in the bone marrow (containing 5.2±2.3% GFP+ cells; n = 10) and spleen (containing 10.7±3.5% GFP^+^ cells; n = 10) of leukemic mice was evaluated by flow cytometry. The majority of GFP+ tumor cells expressed CD48 (99.3±0.7%), CD47 (89.5±6.9%) and Mac1 (74.4±14.7%), while expression of Gr1 (4.73±2.65%), B220 (1.35±1.52%), CD4 (18.6±16.8%), and Ter119 (3.13±1.6%, Fig. 1E) was low or absent. Variable levels of CD8 (68.8±14.9%) and CD71 (58.6±16.8%) were seen in a subset of mice (10 out of 32 animals examined). Although these data indicate substantial heterogeneity in the leukemic clones propagated *in vivo*, this antigen expression pattern is consistent overall with an acute leukemia of myeloid phenotype.

### Injection of p16p19*^−^* ^/*−*^; Kras(G12V) bone marrow cells in the hindlimb induces localized histiocytic sarcoma

To model murine histiocytic/myeloid sarcoma in NOD.SCID mice, we adopted an intramuscular transplantation system previously established to induce rhabdomyosarcomas in skeletal muscle [24]. The same donor cell population as in the retro-orbital injection experiments described above was injected into the gastrocnemius muscles of NOD.SCID mice following pre-injury with cardiotoxin (n = 46 individual mice, 5 independent experiments; Table 1). 39 out of 46 animals receiving *p16p19^−/−^; Kras(G12V)* BM cells developed tumors at the site of injection within 30–50 days (one additional mouse developed tumor at day 70, Table 1, Fig. S1). In parallel control experiments, NOD-SCID mice receiving *p16p19^−/−^; Ctrl* BM cells (n = 16 mice, 3 independent experiments) or *WT; Kras(G12V)* BM cells (n = 10 mice, 2 independent experiments) in the cardiotoxin pre-injured gastrocnemius muscles produced no tumors (Table 1), nor did C57BL/6 mice receiving *p16p19^−/−^; Kras(G12V)* BM cells in pre-injured muscles (0/10). To assess the influence of cardiotoxin pre-injury on subsequent tumor development, *p16p19^−/−^; Kras(G12V)* BM cells were injected into the gastrocnemius muscles of an additional cohort of NOD.SCID mice without cardiotoxin pre-injury (n = 10 mice, 2 independent experiments, Table 1). Tumor frequency and latency were found to be independent of cardiotoxin pre-injury (tumors developed in 84.8% of muscles pre-injured by cardiotoxin injection and in 80% of uninjured muscles, p = 0.68).

**Table 1:** Summary of intra-muscular injection in NOD.SCID mice.

Groups	Recipient strain	No. of cells transplanted	Tumor onset time(days)	Cardio-toxin Pre-injury	Tumor/recipients
Ink4A/Arf^−/−^; KrasG12V Primary Transplant					
p16p19^−/−^; KrasG12V	NOD.SCID	5×10^6^	30–50, 70	Y	39/46
p16p19^−/−^; Ctrl	NOD.SCID	5×10^6^	N/A	Y	0/16
WT; KrasG12V	NOD.SCID	5×10^6^	N/A	Y	2/10
p16p19^−/−^; KrasG12V	NOD.SCID	5×10^6^	60–70	N	8/10
p16p19^−/−^; KrasG12V	C57/B6	5×10^6^	N/A	N	0/10
Secondary Transplant (GFP+ cells)					
Group 1	NOD.SCID	100 K	24–40	Y	12/12
Group 2	NOD.SCID	10 K	25–40	Y	10/10
Group 3	NOD.SCID	2 K	50	Y	1/6
Group 4	NOD.SCID	200	N/A	Y	0/2
MLL-AF9 tertiary transplant					
MLL-AF9 2^nd^ BM cells	NOD.SCID	1×10^6^	∼16	Y	15/15
Ds-Red control BM cells	NOD.SCID	1×10^6^	N/A	Y	0/5

Data are compiled for the three sets of experiments described in the text, including primary transplantation using *p16p19^−/−^; Kras(G12V)* BM cells, secondary transplantation using GFP^+^ sorted tumor cells, and tertiary intra-muscular injection using *MLL-AF9/DsRed* leukemia BM cells. Data include source of cells used for transplant, number of cells transplanted, time of tumor onset (days) after initial injection, whether the mice received cardiotoxin pre-injury, and percentage of mice developing tumors among all recipients.

**Figure 2:**
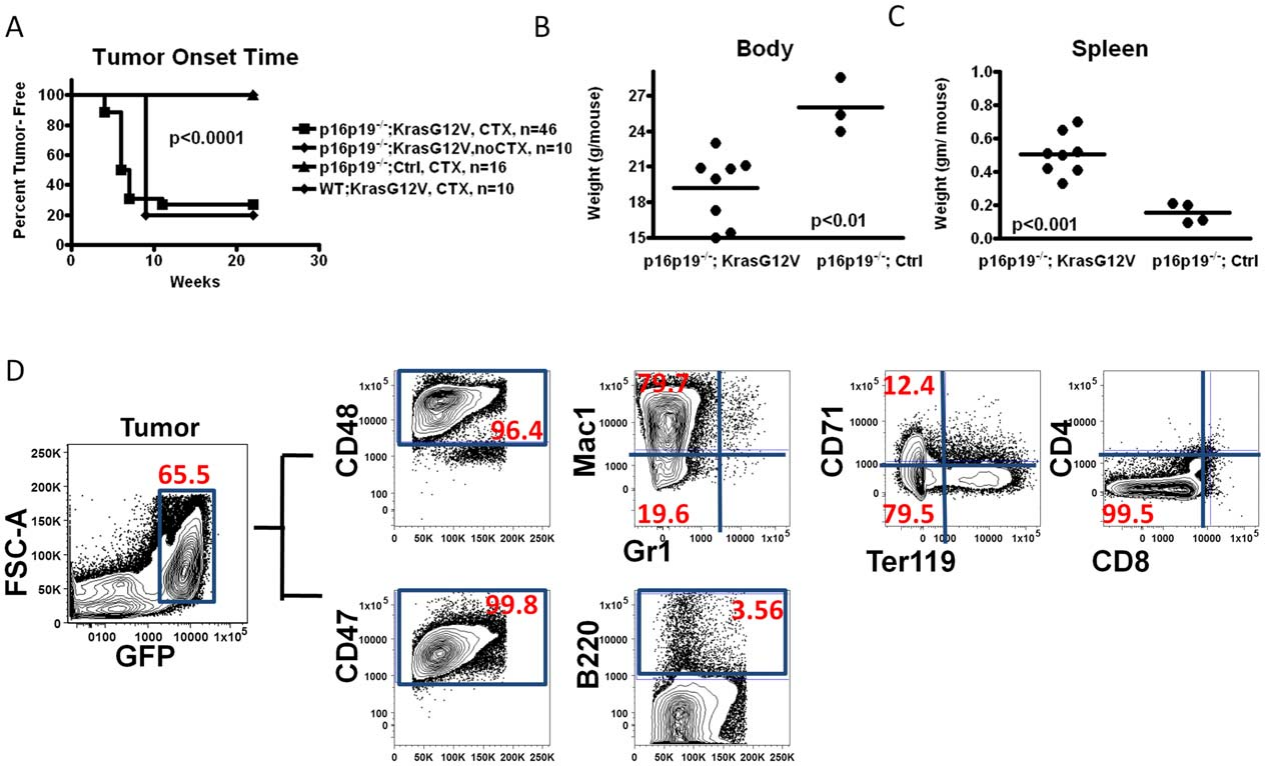
Identical leukemogenic system generates histiocytic sarcoma in the hind limb of NOD.SCID mice when injected into the gastrocnemius muscle. (A) Kaplan-Meier curve showing the fraction tumor-free mice at the indicated time after transplant of *p16p19^−/−^; Kras(G12V)-GFP* with or without cardiotoxin (CTX) pre-injury, or of *p16p19^−/−^ ;Ctrl* or *WT; Kras(G12V)* cells, with CTX pre-injury, into the muscles of NOD.SCID mice. (B, C) Body weight and spleen weight analyses at the time of sacrifice for NOD.SCID mice receiving *p16p19^−/−^; Kras(G12V)* or *p16p19^−/−^ ;Ctrl* intra-muscular transplantation. Mice were sacrificed approximately 2 weeks after initial tumor detection. (D) Flow cytometry analysis of a representative tumor sample from a NOD.SCID mouse bearing an *p16p19^−/−^; Kras(G12V)-GFP* muscle tumor, revealing that most GFP^+^ tumor cells (gating shown on leftmost plot) are CD48^hi^, CD47^hi^, Mac1^hi^, but Gr1*^−^* ^/lo^, B220*^−^* ^/lo^, CD4*^−^* ^/lo^, CD8*^−^* ^/lo^, CD71*^−^* ^/lo^ and Ter119*^−^* ^/lo^.

Phenotypic analyses of tumor samples indicated that tumors arising from *p16p19^−/−^; Kras(G12V)* BM cells were comprised mostly of immature GFP^+^ monocytic cells (61.2±13.9%, Fig. 2D). The immunophenotype of GFP^+^ tumor cells recovered from muscle was highly similar to that of leukemic cells recovered from mice transplanted retro-orbitally with *p16p19^−/−^; Kras(G12V)* cells (Fig. 1E), including high levels of CD48 (96.77±3.6%), CD47 (81.09±16.30%), and Mac1 (79.59±18.91%), and low to absent expression of CD4 (26.52±25.54%), B220 (16.77±16.4%), Ter119 (15.5±14.5%), CD71 (30.1±15.4%), Gr1 (7.54±8.4%), and CD8 (9.24±9.05%) (Fig. 2D). One notable exception was the more frequent expression of CD8 by tumor cells recovered from the bone marrow of mice transplanted retro-orbitally with *p16p19^−/−^; Kras*-transduced cells (68.8±14.9% vs. 9.24±9.05%, Figs. 1E and 2D). In general, however, the immunophenotype of the murine histiocytic neoplasm induced from *p16p19^−/−^; Kras(G12V)* BM cells appears to be independent of its primary site and particular microenvironment.

**Figure 3:**
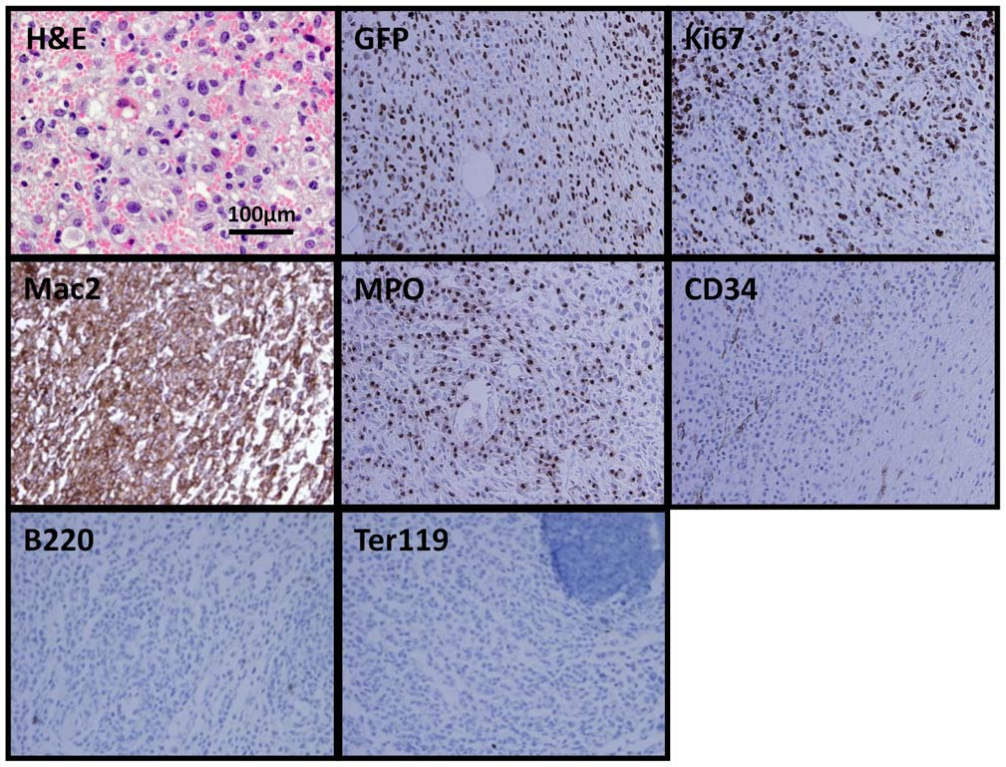
Histopathological examination of histiocytic sarcoma tissue sections. Hematoxylin & eosin staining of tumor (60x) indicating that myeloid cells comprise a majority of the tumor cells. Immunohistochemistry staining of tumor samples shows positive staining for GFP, Ki67, Mac2, and MPO, and negative staining of the non-myeloid hematopoietic lineage markers, B220 and Ter119 (40x).

Microscopic examination of tumor sections revealed extensive infiltration of tumor cells into the muscle and surrounding adipose tissue, with nearly complete destruction of normal muscle architecture. Infiltrating cells were immature-appearing intermediate to large size cells with round or irregular nuclei, prominent nucleoli and moderate to large amounts of cytoplasm (Fig. 3). Scattered macrophages also were noted. Immunohistochemical staining of primary tumor samples demonstrated that the tumor cells were GFP^+^ (consistent with flow cytometry analyses (Fig. 2D) and with derivation from cells infected by *Kras(G12V)-GFP* virus) and stained strongly for Mac2, consistent with a myeloid phenotype. Tumors lacked expression of CD34, B220 and Ter119 (Fig. 3), but showed clearly detectable expression of myeloperoxidase (MPO), which marks myeloid cells. Finally, consistent with their aggressive growth kinetics, tumors exhibited extensive reactivity for the cell proliferation marker Ki67 (detected in 51.9±8.8% of all hematoxylin counter-stained cells, n = 8 slides) (Fig. 3). Based on their morphology and staining pattern, the muscle tumors arising from *p16p19^−/−^; Kras(G12V)* BM cells were classified as histiocytic sarcomas (equivalent of human myeloid sarcomas).

### Dissemination of histiocytic sarcoma cells

**Figure 4:**
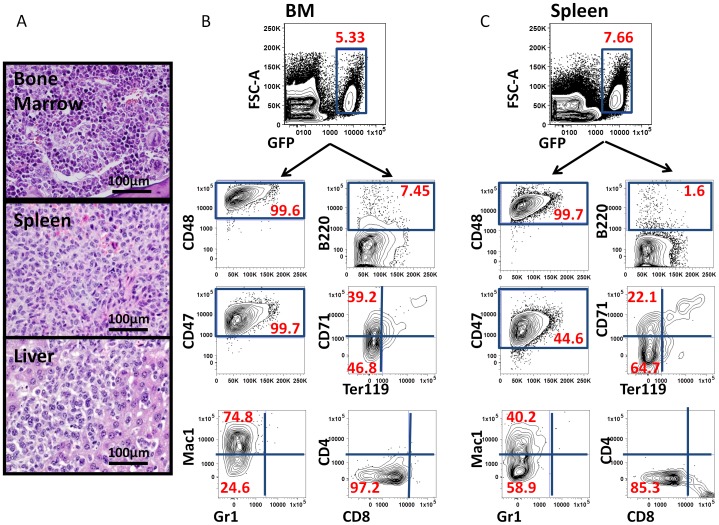
Phenotypic profiling of bone marrow and spleen cells indicates dissemination of tumor cells from hindlimb muscle into hematopoietic organs. (A) H & E staining of bone, spleen and liver from recipients bearing primary myeloid tumor in the muscle, indicating aggressive infiltration of myeloid leukemia cells (60x) to distant anatomical locations. (B, C) Representative flow cytometry data demonstrating a similar immunophenotype of tumor cells in BM (B) and spleen (C) as that seen in the primary histiocytic sarcoma initiated in muscle (see Fig. 2D).

Clinically, myeloid sarcomas often coincide with or precede the diagnosis of new or recurrent acute myeloid leukemia [31]. Review of peripheral blood cell morphology of tumor bearing mice revealed polychromasia and marked reticulocytosis consistent with extramedullary hematopoiesis, as well as scattered immature monocytic cells, consistent with monoblasts, in 2 animals (data not shown). We also examined non-muscle hematopoietic tissues for the presence of GFP^+^ tumor cells. Nests of myeloid-appearing cells were noted in the bone marrow, spleen and liver of tumor-bearing animals (Fig. 4A). Enlarged spleens were observed in all tumor-bearing mice (Fig. S1B). The immunophenotype of GFP^+^ cells in the bone marrow and spleen of sarcoma-bearing mice was evaluated by flow cytometry. GFP^+^ cells were CD48^hi^ (95.4±3.1% in bone marrow, 98.9±0.9% in spleen), CD47^hi^ (93.4±6.1% in bone marrow, 89.2±11.7% in spleen), Mac1^mid/hi^ (79.1±12.5% in bone marrow, 70.5±17.9% in spleen), and B220^−/lo^, CD4^−/lo^, CD8^−/lo^, Ter119^−/lo^, and CD71^lo/mid^ (Fig. 4B, 4C). This immunophenotype is highly consistent with the phenotype of GFP+ cells isolated from primary histiocytic sarcomas induced by intramuscular injection of *p16p19^−/−^; Kras(G12V)* BM cells (see Fig. 2D), as well as the phenotype of GFP^+^ leukemia cells derived by retro-orbital injection of BM cells modified by the same oncogenetic lesions (see Fig. 1E). Thus, induction of histiocytic sarcomas in skeletal muscle ultimately results in systemic dissemination with involvement of bone marrow, spleen and liver, thereby recapitulating the natural course of myeloid sarcomas in humans preceding manifestation of systemic leukemias [30].

### Histiocytic sarcoma is highly transplantable

**Figure 5:**
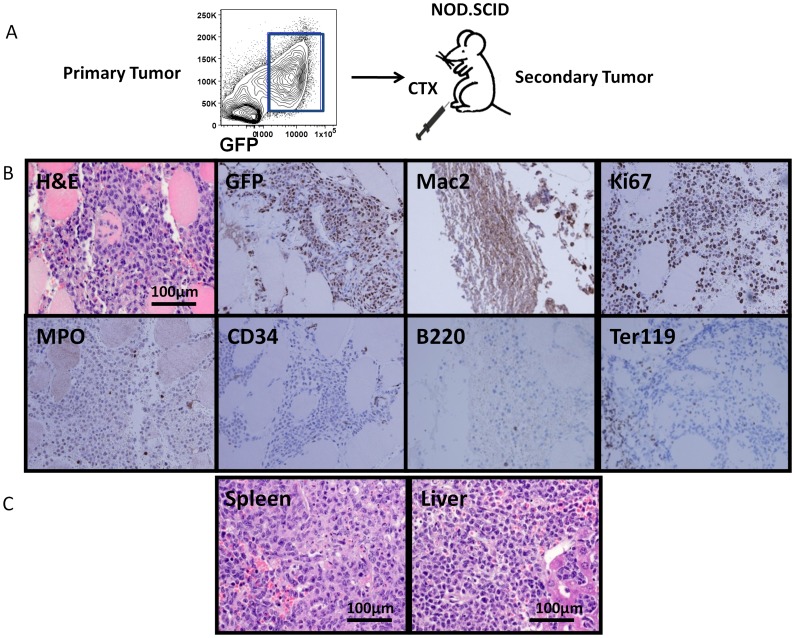
Primary histiocytic sarcomas are serially transplantable in NOD.SCID mice. (A) Schematic diagram showing experimental design for secondary transplantation. GFP^+^ cells were sorted from freshly isolated primary tumor, induced initially by transplantation of gene-modified bone marrow cells into the cardiotoxin pre-injured muscle of NOD.SCID recipients, and injected into gastrocnemius muscle of secondary NOD.SCID recipients at different cell dosages (100 000, 10 000, 2 000, or 200 cells) following cardiotoxin pre-injury. (B) H & E staining of tumor cells (60x) in skeletal muscle of a secondary recipient. Immunohistochemical staining of secondary tumors shows positive staining for the myeloid marker Mac2, and negative staining of B220 and Ter119 (40x). (C) H & E staining of liver and spleen from secondary recipient mouse, showing aggressive infiltration of myeloid leukemia cells (60x).

The rapid growth of *p16p19^−/−^; Kras(G12V)* histiocytic sarcoma suggested that the initial seeding cell population contained clones with leukemia-propagating ability. To test directly the tumor-propagating potential of *p16p19^−/−^; Kras(G12V)* histiocytic sarcomas, GFP^+^ cells from primary tumors induced in the muscles of primary NOD.SCID recipients were sorted using FACS and defined numbers of sorted cells were transplanted into the cardiotoxin pre-injured gastrocnemius muscle of secondary NOD.SCID recipients (Fig. 5A). The frequency of tumor-propagating cells within the GFP+ population was assessed by limiting dilution analysis (using 200 to 100 000 cells per injection). All mice receiving 100 000 (12/12 mice injected, 3 independent experiments) or 10 000 (10/10 mice injected, 2 independent experiments) GFP+ primary sarcoma cells developed tumors within 25–40 days from injection (Table 1, Fig. S1C). These studies suggest that the latency of secondary tumor formation is markedly shorter than that of primary tumor formation (30–50 days for tumor induction with 5×106 *p16p19−/−; Kras(G12V)* BM cells, Table 1). The smallest number of GFP+ cells giving rise to a histiocytic sarcoma in this analysis was 2 000 cells (1/6 mice transplanted, 3 independent experiments), which yielded a tumor 50 days after transplant (Table 1). No tumors were detected in recipients receiving 200 GFP+ cells (0/2 mice transplanted, Table 1). Based on these data, the average frequency of tumor-propagating cells in *p16p19^−^/−; Kras(G12V)* histiocytic sarcomas is 1/3 765 (confidence choice 95%, confidence intervals 1 870–7 578).

Secondary tumors exhibited histology similar to that of primary tumors and were GFP^+^ and Mac2^+^ by immunohistochemistry (Fig. 5B). Weak staining for MPO was detected among tumor samples (Fig. 5B). Similar to primary tumor-bearing mice, the livers and spleens of secondary recipients showed extensive involvement by infiltrating myeloid cells (Fig. 5C, Fig. 4A). Secondary tumors showed a high proliferative index (80±16.4% Ki67^+^ of all hematoxylin counter-stained cells, n = 5), and peripheral blood smears lacked involvement by histiocytic sarcoma (data not shown). Thus, *p16p19^−/−^; Kras(G12V)* induced histiocytic sarcomas are highly transplantable in vivo.

### Primary histiocytic sarcomas are oligo-clonal

**Figure 6:**
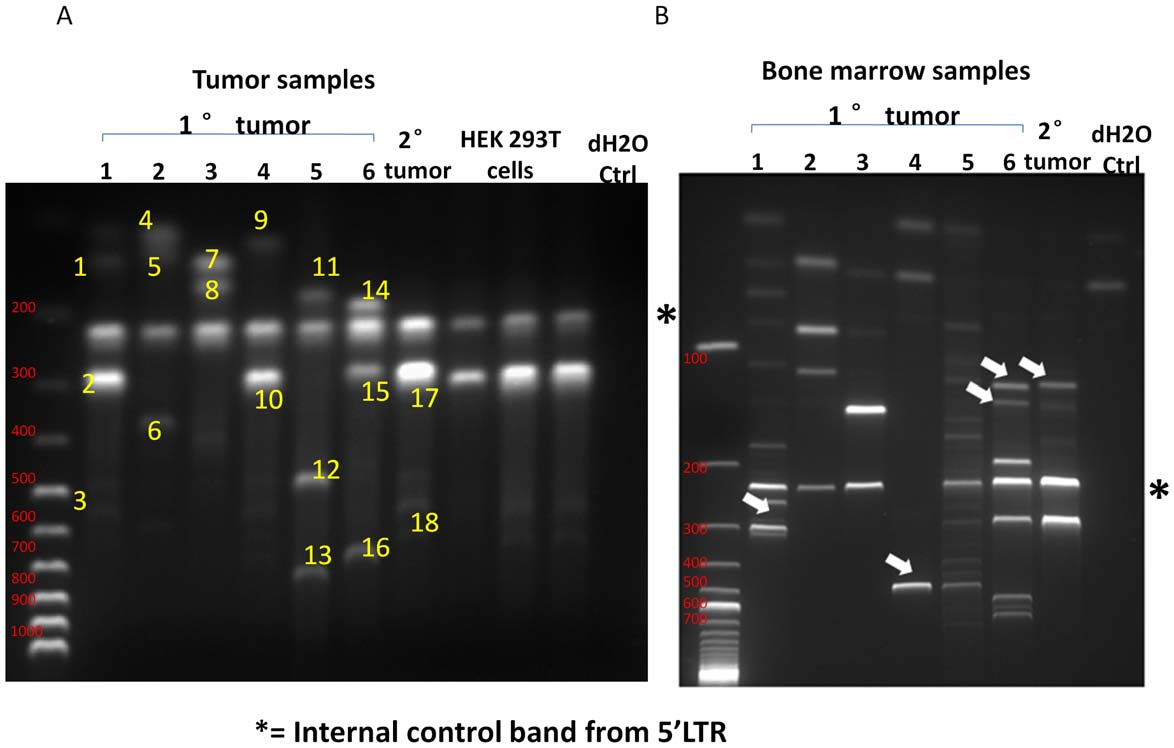
Clonality analysis for genomic DNA samples extracted from primary and secondary histiocytic sarcomas and BM samples. (A) Genomic DNA was extracted from primary and secondary tumors, and subjected to LM-PCR assay. Electrophoretic results of the final products are shown. Numbers at top of gel refer to sample numbers: No. 1 to 6 are primary tumors, No. 7 is a secondary tumor transplanted using GFP^+^ tumor cells from sample No. 4. All tumors possess an average of three integration sites, demonstrated by distinct numbers and sizes of bands (yellow numbers on the gel). HEK293T cells were transduced with the same virus and subject to LM-PCR as control. Size markers (100 bp ladder, size of individual bands indicated in red text) shown at left. (B) Genomic DNA was extracted from BM samples of the same set of NOD.SCID mice bearing primary and secondary tumors as analyzed in (A). DNA was subjected to LM-PCR assay. Electrophoretic results of the final products are shown. All BM samples possess an average of three to four integration sites. Bands unique of bone marrow samples are indicated by white arrowheads. Size markers (100 bp ladder) shown at left.

The lentivirus-based strategy we employed to generate hematopoietic tumors has the inherent advantage that the transformed cells are uniquely marked by viral integration [32], allowing direct assessment of tumor clonality. We therefore asked if the *p16p19^−/−^; Kras(G12V)* histiocytic sarcomas were initiated by a single clone or multiple clones of tumorigenic cells by analysis of proviral integration sites. Genomic DNA was extracted from both primary and secondary histiocytic sarcomas as well as from the BM of the same set of tumor-bearing mice and subjected to Ligation-Mediated PCR (LM-PCR) Assay [33]. Three PCR bands, on average, were observed in each of the primary tumor samples, indicating tumor oligoclonality (Fig. 6A). Sequencing of PCR bands identified distinct loci in the murine genome, thus validating that the LM-PCR products indicated *bona fide* proviral integration sites (Fig. 6A, Fig. S2). Of note, sequencing of LM-PCR products demonstrated that a single clone harboring an insertion in the proximity of Ribosomal Protein S29 (RPS29) was present in one set of matching primary and secondary transplanted tumors (bands 10, 17, 18, Fig. 6 and Fig. S2). These data suggest that a dominant clone of the tumorigenic cell population may have been selected during serial transplantation, explaining the shortened tumor latency in secondary transplant recipients. We also compared integration bands identified in tumors and bone marrow specimens from 7 individual mice. 7 out of 7 BM samples were polyclonal, and appeared to share subsets of the dominant clones with the muscle-resident tumors (Fig. 6B). However, we also detected additional novel bands in 4 out of 8 bone marrow samples (Fig. 6B, marked by white arrows), suggesting differences in selection forces on tumor cells in different microenvironments.

### Histiocytic sarcoma can be induced using alternative leukemogenic strategies

The very high rate of induction of histiocytic sarcoma observed using *p16p19^−/−^; Kras(G12V)* BM cells as a donor cell population prompted us to ask whether this might be an intrinsic property of this oncogenic combination, or whether histiocytic sarcomas can be introduced in NOD.SCID mice using other leukemogenic systems. We therefore acquired a leukemic cell sample in which hematopoietic malignancy was induced using the *MLL-AF9* retroviral system [34]. *MLL-AF9*-expressing cells, marked by co-expression of Ds-Red, were harvested from secondary leukemic BM and transplanted into the pre-injured gastrocnemius muscles of NOD.SCID mice (Fig. S3A). All of these tertiary recipients developed tumors in the injected hindlimb within 16 days (15/15 mice injected, Fig. S1D and Table 1), while control mice injected with DS-Red labeled WT BM cells showed no tumor development (0/5 mice injected, Table 1).

It previously has been reported [35] that the *MLL-AF9* retroviral model induces acute myeloid leukemia in mice. *MLL-AF9* tumors in our model were consistently comprised of a dominant DsRed^+^ cell fraction (80.9±3.9%; Fig. S3B, n = 10). Similar to *p16p19^−/−^; Kras(G12V)* histiocytic sarcomas (Fig. 2D) and leukemias (Fig. 1E), *MLL-AF9/DsRed* tumors were CD48^hi^ (95.8±2.4%), CD47^hi^ (95.3±2.9%), Mac1^hi^ (83.4±7.8%), Gr1^mid/lo^ (20.2±2.4%), and B220*^−^* ^/lo^, CD4*^−^* ^/lo^, CD8*^−^* ^/lo^, Ter119*^−^* ^/lo^ (Fig. S3B, n = 10). DsRed^+^ cells also spread to distant hematopoietic organs, including the BM and spleen, after tumor onset (Fig. S3C). Of note, the percentage of DsRed^+^ cells in both the spleen and marrow markedly increased within 10 days, less than one week after formation of palpable tumors at the injection site in muscle (BM: 2.10±1.75% DsRed^+^ cells increased to 48.38±3.28%; spleen: 2.89±0.35% DsRed+ cells increased to 46.85±5.18%, Fig. S3C and data not shown, 10 out of 15 animals examined). Thus, as in the *p16p19^−/−^; Kras(G12V)* model, in the *MLL-AF9* model, systemic development of myeloid leukemia in recipient mice occurs following extramedullary tumor formation, with fluorescently marked tumor cells migrating from the injection site in the hindlimb muscle to the BM and spleen.

## Discussion

The work described here establishes a novel in vivo lentivirus-induced histiocytic/myeloid sarcoma model in immuno-compromised mice, combining ablation of the tumor suppressor gene locus *p16p19* and ectopic expression of constitutively active oncogenic *Kras(G12V)*. *P16p19^−/−^; Kras(G12V)* tumor cells exhibit typical features of histiocytic sarcoma, including a predominant lack of expression of lymphocyte markers, positive expression of histiocyte/macrophage markers, and round to oval shaped cells with abundant, eosinophilic cytoplasm and nuclear atypia. *P16p19^−/−^; Kras(G12V)* induced murine histiocytic sarcomas are aggressive neoplasms, and all tumor-bearing mice ultimately succumb to progressive leukemic symptoms [36].

The events that drive the formation of murine histiocytic sarcomas versus murine histiocytic leukemias as the first manifestation of a histiocytic neoplasm remain unclear. Murine histiocytic tumors induced by *P16p19^−/−^; Kras(G12V)* tumor cells show evidence of a monocytic origin. A recent report showed that mice with coincident loss of *Dok-1*, *Dok-2* and *Dok-3* genes develop highly invasive and transplantable histiocytic sarcoma endogenously, and *Dok-1/2/3^−^* ^/*−*^ macrophages demonstrate enhanced proliferation ability [37], suggesting origination of the disease in monocytic cells. Histiocytic sarcoma also has been observed sporadically in *pEμ-Ras* transgenic mice [38], and *p16p19^−/−^* mice develop histiocytic sarcoma with homozygous loss of *Pten* [39]. Deficiency of *Pten* leads to activation of Akt, as well as ERK1 and ERK2 in the histiocytic sarcoma cells, indicating hyperactivation of the Kras-MAPK pathway [39]. The majority (75%) of *Pten^−^* ^/*−*^ *p16p19^−/−^* mice show a biphasic pattern of both lymphoblastic lymphoma (with a predominance of B- over T-cell lymphoma) and histiocytic sarcoma [39]. In this regard, it is intriguing that in our study, we observed variable expression of the lymphoid marker CD8 in a subset of leukemic mice transplanted intravenously with *p16p19^−/−^; Kras(G12V)* cells, whereas CD8 was less frequently expressed in histiocytic sarcomas generated by intramuscular transplantation of the same donor cell population. These data reinforce the previously suggested association of lymphomatous disease with histiocytic sarcoma [39], and suggest that the anatomical location of tumor origination may influence the manifestation of this biphenotypic pattern.

P16P19 proteins typically act as tumor suppressors for T cell and B cell malignancies [28], while *Kras* mutation induces myeloid leukemias [13]. Of note, in both the *p16p19^−/−^*; *Kras(G12V)* and *MLL-AF9* sarcoma models studied here, BM cells were cultured with a cytokine cocktail briefly (3 hr) after isolation and prior to intramuscular injection (see *Materials and Methods*). We cannot rule out the possibility that selective pressure from the cytokines possibly confers a myeloid bias on the engrafting cells [40]. Nonetheless, given prevalent *in vivo* data from murine leukemia models endogenously expressing either of the oncogenic combinations employed here [25], [41]–[43], it is reasonable to expect that culture conditions had only a minor impact on the lineage decision of the histiocytic tumor-initiating cells.

These studies demonstrate clear similarities between murine histiocytic sarcoma and murine histiocytic leukemia cells established by injection of the same oncogenetically modified BM cells in distinct anatomical locations (muscle vs. blood). A growing number of studies indicate that hematopoietic and leukemic cells functionally interact with a number of different “niche” cells in the BM, e.g., osteolineage cells, mesenchymal cells, reticular cells, endothelial cells, and adipocytes, as reviewed in [44]. These bi-directional interactions are mediated by an array of molecular and cellular signaling pathways, such that perturbations in microenvironmental regulators can influence both normal hematopoiesis and leukemic progression [45]. In particular, Wei et al reported that the lineage fate of the human *Mll-AF9* leukemia cells in mice could be altered by manipulating growth signals or recipient strain [46]. Furthermore, mice with AML induced by co-expression of BCR/ABL and the Nup98/HoxA9 fusion protein showed a loss of osteolineage cells in the marrow, which may have contributed to the underlying pancytopenia [47]. Finally, “niche”-specific deletion of the microRNA processing enzyme Dicer (using conditional ablation in mouse osteolineage cells) was shown to be sufficient to drive the development of a hematopoietic malignancy that requires this altered microenvironment for its continued propagation [48]. Yet, despite clear evidence of functional cross-talk between leukemic and niche cells in the marrow, much remains to be discovered about the role of the “niche” in the initiation and progression of extramedullary hematopoietic malignancy *in vivo*. Interestingly, the murine histiocytic sarcoma/leukemia models described here indicate a dominant influence of cell-autonomous, as opposed to microenvironmental, signals in the development of these malignancies. Whether arising initially in the skeletal muscle or hematopoietic tissue, the histiocytic sarcoma and leukemia cells induced by *p16p19* deletion and *Kras* activation share nearly identical morphological, phenotypic, and histopathological features. Moreover, both neoplasms progress to systemic disease with similar kinetics and dissemination patterns. These findings de-emphasize possible cell-non-autonomous effects of the microenvironment in which tumors are initiated as critical determinants of disease phenotype or progression, and highlight instead the strong influence of a cell-autonomous oncogenic program in specifying the emergence of these hematopoietic tumors.

The histiocytic sarcomas and leukemias reported here uniformly express high levels of CD47. Also known as Integrin Associated Protein (IAP), CD47 acts as a “don't eat me” signal, such that cells with high surface expression of CD47 escape integrin-mediated phagocytosis and death [49]. As previously reported, circulating hematopoietic stem cells, human and mouse myeloid leukemia cells, human bladder tumor-initiating cells, and multiple myeloma cells all express CD47 at an elevated level, and antagonistic treatment with anti-CD47 antibody both in vivo and ex vivo can induce remission of these cancers in mouse and xenograft models [50]–[53], suggesting that strategies targeting CD47 may provide promising therapeutic avenues in a variety of malignancies, regardless of the underlying oncogenetic lesions. As the histiocytic (myeloid) sarcomas studied here included a majority population of CD47^+^ tumor cells, the detection and targeting of CD47 in these tumors may present a useful therapeutic target. Related to this, recent studies have identified >20 distinct amino acid and glycosylation differences in the CD47 ligand SIRP-α (signal regulatory protein-α) in NOD.SCID mice (as compared to other mouse strains, including C57BL/6) [54]. SIRP-α is expressed by macrophages and inhibits their phagocytic function when bound by CD47 [55]. Protein coding as well as post-translational polymorphisms in SIRP-α correlate directly with the higher engraftment capacity in NOD.SCID mice of transplanted human hematopoietic stem cells [54]. Based on these findings, it is tempting to speculate that differences in the capacity for or consequences of recognition of tumor cell-expressed CD47 by SIRP-α-expressing macrophages in NOD.SCID versus C57BL/6 mice may contribute to the ability of intra-muscularly injected *p16p19^−/−^*; *Kras(G12V)* tumor cells to generate histiocytic sarcomas in NOD.SCID but not C57BL/6 recipients, as reported here.

In summary, this work indicates that study of histiocytic (myeloid) sarcoma need not be limited to the few spontaneously emergent models currently available [37]–[39]. Through direct ex vivo modification and transplantation of oncogene-modified hematopoietic lineage cells, we established a rapid and reproducible system for the generation of this extramedullary tumor, and showed that this model recapitulates the natural progression of the disease in leukemia patients. Moreover, the tumor cells share highly similar features irrespective of the microenvironment at the site of initiation. Future studies using this model to uncover the molecular mechanisms that drive the establishment and dissemination of these malignancies will aid in the rapid diagnosis and effective treatment of these high risk hematopoietic tumors.

## Materials and Methods

### Mouse husbandry and breeding

6–10 week old NOD.CB17-Prkdcscid/J (NOD.SCID) mice (JAX, Bar Harbor, Maine) and *p16p19^−/−^* mice (B6.129 background, NIH/Mouse Models of Human Cancer Consortium) were bred and maintained at the Joslin Diabetes Center Animal Facility.

This study was carried out in strict accordance with the recommendations in the Guide for the Care and Use of Laboratory Animals of the National Institutes of Health. All animal experiments were approved by the Joslin Diabetes Center Institutional Animal Care and Use Committee (Protocol 04-01 to Amy Wagers). All surgeries were performed under anesthesia, and all efforts are made to minimize suffering. Animals were humanely sacrificed prior to tissue collection.

### Preparation of lentivirus

The *Kras(G12V)*-IRES-GFP pGIPZ plasmid was a gift from Dr. Junhao Mao (University of Massachusetts, Worcester, MA). The *Kras(G12V)*-IRES-GFP pGIPZ plasmid (10 µg) or pGIPZ vector plasmid (10 µg, Open Biosystems, Rockford, IL), the HIV gag-pol-REV expression plasmid pCMV-dR8.91 (6.5 µg) and the envelope expression plasmid pMD2.VSV.G (3.5 µg) were co-transfected into 293 T cells with Fugene 6 (Roche Indianapolis, IN) on Day 1. HEK 293T cell line was a gift from former Gary D. Gilliland's lab [56]. Medium was changed every day for the next 3 days, and supernatant collected on Days 3 and 4. Supernatant was concentrated by ultracentrifugation at 20,000 rpm for 3 hours at 4°C (Beckman L7 ultracentrifuge). Lentivirus was titrated and then stored at −80°C for ≤3 weeks prior to use. Titration of lentivirus was performed using 293 T cells. The percentage of GFP+ cells at 72 hrs post infection was determined using flow cytometry.

### Lentiviral transduction of bone marrow cells

Freshly isolated bone marrow (BM) cells were plated in Iscove's Modified Dulbecco's Medium (IMDM) (31980, Gibco, Grand Island, NY) containing 5% fetal bovine serum (FBS), 1% penicillin/streptomycin, 200 mM glutamine, 1% non-essential amino acids, 1% sodium pyruvate, 50 μM 2-mercaptoethanol, stem cell factor (SCF, 10 ng/ml, Peprotech, Rocky Hill, NJ), Flt3-L (10 ng/ml, Peprotech), IL-11 (10 ng/ml, Peprotech), thrombopoietin (TPO, 10 ng/ml, Peprotech), IL-6 (10 ng/ml, Peprotech), and IL-3 (10 ng/ml, Peprotech). Bone marrow cells were spin-infected with 8 μg/ml polyprene and lentivirus (MOI of 0.1–0.5), at 2,500 rpm for 90 min. BM cells were then incubated for 3 hours at 37°C, counted and injected into NOD.SCID mice.

### Tumor induction

Lentivirally transduced cells were washed and resuspended in Hank's balanced salt solution (HBSS) containing 2% FBS. Cells were injected retro-orbitally using a 28G 1/2 insulin syringe, or into the gastrocnemius muscles of isofluorane anesthetized NOD.SCID mice using a transdermally inserted dental needle attached to a Hamilton syringe via polyethylene tubing. Recipient muscles were pre-injured 24 hours before cell implantation by injection of 25 μl of a 0.03 mg/ml solution of cardiotoxin (from *Naja mossambica*, Sigma, St. Louis, MO). Muscle pre-injury has been shown in previous studies [57] to enhance the engraftment of transplanted muscle precursor cells, although pre-injury was not required for induction of murine histiocytic sarcoma (see Table 1).

For secondary transplantation, tumor tissue was harvested from tumor-bearing mice, dissociated to generate single cell suspensions, and then GFP^+^ sarcoma cells were isolated by Fluorescence Activated Cell Sorting (FACS) (see Cell Isolation Procedures for additional information). Recovered cells were suspended in HBSS with 2% FBS and injected into the cardiotoxin injured gastrocnemius muscles of secondary recipient mice, as described above.

### Histology/Immunohistochemistry

Tissues were fixed in 4% paraformaldehyde for 3–5 hours and embedded in paraffin. Sections (4 micron) were stained with Hematoxylin & Eosin (H&E) or subjected to immunohistochemistry staining at the Specialized Histopathology Core Facility of the Dana-Farber/Harvard Cancer Center using the following antibodies: GFP (1∶1500 in EDTA, ab6556, Abcam, Cambridge, MA), Ki67 (1∶250 in EDTA, VP-RM04, Vector Labs, Burlingame, CA), CD34 (1∶100 in citrate, ab8158, Abcam), MPO (1∶2000 in EDTA, A0398, Dako, Carpinteria, CA), B220 (1∶200 in citrate, 550286, BD Pharmingen, Franklin Lakes, NJ), Ter119 (1∶2000 in citrate, 550565, BD Pharmingen).

### Cell isolation procedures

Bone marrow cells were flushed from femurs and tibias with HBSS containing 2% FBS. Spleen cells were isolated by mechanical dissociation in HBSS supplemented with 2% FBS. Muscle tumors were digested in DMEM +0.2% collagenase type II (Invitrogen) for 90 minutes at 37°C in a shaking water bath, triturated to disrupt the remaining tumor pieces and filtered through a 70 µm cell strainer. Red blood cells were lysed from all cell preparations using ACK lysing buffer (Lonza, Hopkinton, MA). Cells were resuspended in HBSS with 2% FBS for subsequent procedure and analyses.

### Flow cytometry analysis

Flow cytometry was performed as described [58]. Non-specific antibody binding was blocked with rat IgG (Sigma) for 15 minutes on ice. Antibody staining was performed for 20 minutes on ice. The following antibodies were used: PE-CD4 (100408, BioLegend, San Diego, CA), PECy7-CD8 (100722, BioLegend), APC-Mac1 (17-0112-82, eBioscience, San Diego, CA), APCCy7-Gr1 (108424, BioLegend), APC-Ter119 (116212, BioLegend), PE-CD71 (113808, BioLegend), PE-CD48 (103405, BioLegend), APC-CD47 (17-0471, eBioscience). Prior to FACS, cells were suspended in HBSS containing 2% FBS and 1 mg/ml propidium iodide to mark non-viable cells (which were excluded from analysis).

### Limiting Dilution Analyses

Limiting dilution analyses were performed based on Bonnefoix et al. [59] using the limdil function of the ‘StatMod’ package (author G.K. Smyth, http://bioinf.wehi.edu.au/software/limdil/), part of the R statistical software project (http://www.r-project.org).

### Analysis of Proviral integration sites

Proviral integration sites were isolated using ligation-mediated (LM)-PCR as described [60]. Briefly, 200 ng of genomic DNA was digested with *Tsp*509I (New England Biolabs, Ipswich, MA) and then subjected to linear amplification using the biotinylated primer Lenti LTRI (5′-GAGCTCTCTGGCTAACTAGG-3′). The labeled amplification product was then purified using streptavidin coated paramagnetic Dynabeads (Invitrogen, Carlsbad, CA) and was subsequently subjected to ligation with a blunt-ended linker cassette that had been synthesized by hybridization of the oligonucleotides OC1 (5′-GACCCGGGAGATCTGAATTCAGTGGCACAGCAGTTAGG-3′) and OC2 (5′-CCTAACTGCTGTGCCACTGAATTCAGATC-3′). The ligation product was amplified via two rounds of nested PCR, using the primers Lenti LTRII (5′-AGCTTGCCTTGAGTGCTTCA-3′) and OCI (5′-GACCCGGGAGATCTGAATTC-3′) in the first round of amplification; and Lenti LTRIII (5′-AGTAGTGTGTGCCCGTCTGT-3′) and OCII (5′-AGTGGCACAGCAGTTAGG-3′) in the second round of amplification. Reaction conditions were as described [60]. Amplification products were analyzed by agarose gel electrophoresis. Selected amplification products were isolated using Gel Extraction Kit (Qiagen), and cloned into the pCR2.1- TOPO vector (TOPO TA cloning kit, Invitrogen). Proviral integration sites were subsequently sequenced using M13 forward and reverse primers and the results were aligned against the mouse genome using Basic Local Alignment Search Tool (BLAST) program (http://blast.ncbi.nlm.nih.gov/Blast.cgi).

### Generation and isolation of MLL-AF9 leukemia cells

MLL-AF9 mouse leukemias were generated in Dr. David Scadden's laboratory as described below. Actin-DsRED mice (JAX) were backcrossed for 10 generations onto C57BL/6J mice (JAX). These mice were sacrificed four days after injection with 150 mg/kg 5FU. BM cells were isolated from femurs and tibias, and red blood cells were lysed using ACK lysing buffer (Lonza). Cells were incubated in RPMI supplemented with 20% FBS, 1% Pen-Strep, 6 ng/ml of IL-3 (Peprotech), 10 ng/ml of TPO (Peprotech), 10 ng/ml of IL-6 (Peprotech), at 37°C 5% CO2 overnight. MLL-AF9 was introduced by spin-infection (1 000 g for 90 minutes) using a retroviral vector (MSCV-MLL-AF9-neo) [35] in the presence of 8 μg/ml polybrene (Millipore, Billerica, MA). Virus-containing media was removed 4 hours after spin-infection, and 4×10^6^ live cells per mouse were injected into lethally irradiated (9Gy) C57BL/6 recipient mice 12 hours after viral infection. The mice were sacrificed once they became moribund. BM cells were isolated as described above, and subjected to ACK lysis. 200 000 cells were injected into sublethally irradiated (4.5 Gy) C57BL/6 recipients. Once these mice were moribund, ACK-lysed live BM cells were isolated as described above and 1×10^6^ cells/mouse were used for intramuscular injections.

## Supporting Information

Figure S1 Tumorigenesis in NOD.SCID recipients of *p16p19^−/−^; Kras(G12V)* BM cells. (A) Gross anatomy of primary histiocytic sarcoma in the hind-limb of a recipient NOD.SCID mouse. (B) Enlarged spleen from a NOD.SCID mouse bearing primary histiocytic sarcoma. (C) Representative image of spleen and tumor of a NOD.SCID mouse bearing secondary histiocytic sarcoma. (D) Representative picture of NOD.SCID mice bearing MLL-AF9 leukemic BM cell- induced histiocytic sarcoma.
(TIF)

Figure S2 List of viral integration sites detected by LM-PCR of the histiocytic sarcoma samples from NOD.SCID mice in Fig. 6. A common integration site near the RPS29 locus is highlighted in red. Interestingly, a recent genetic screen revealed that mutation of RPS29 abolishes definitive hematopoiesis in zebrafish embryos (Burns CE et al, 2009), and loss of RPS29 affects the expression of hemoglobin suggesting a defect in red blood cells differentiation or hemoglobinization (Taylor AM et al, 2012).
(TIF)

Figure S3 *Ds-Red/MLL-AF9* leukemia cells induce histiocytic sarcoma in intramuscularly-transplanted NOD.SCID recipients. (A) Transplantation schematics for *Ds-Red/MLL-AF9* transplantation. (B) Representative immunophenotypic profiling of *MLL-AD9* induced histiocytic sarcomas. Plots at right are gated for live (PI-) DsRed^+^ cells. (C) Representative frequency of DsRed^+^ cells in bone marrow and spleen of mice in which histiocytic sarcomas were induced by transplantation of *Ds-Red/MLL-AF9*-expressing BM cells. Data collected by flow cytometry at 30 days post transplantation. (D) H & E staining of *Ds-Red/MLL-AF9*-induced histiocytic sarcoma, bone marrow, spleen and liver (60x).
(TIF)
