# Supplementary figures and images for: Induction of Histiocytic Sarcoma in Mouse Skeletal Muscle
Source: PLoS One. 2012 Aug 31;7(8):e44044. doi: 10.1371/journal.pone.0044044 (PMC3432091; doi:10.1371/journal.pone.0044044)

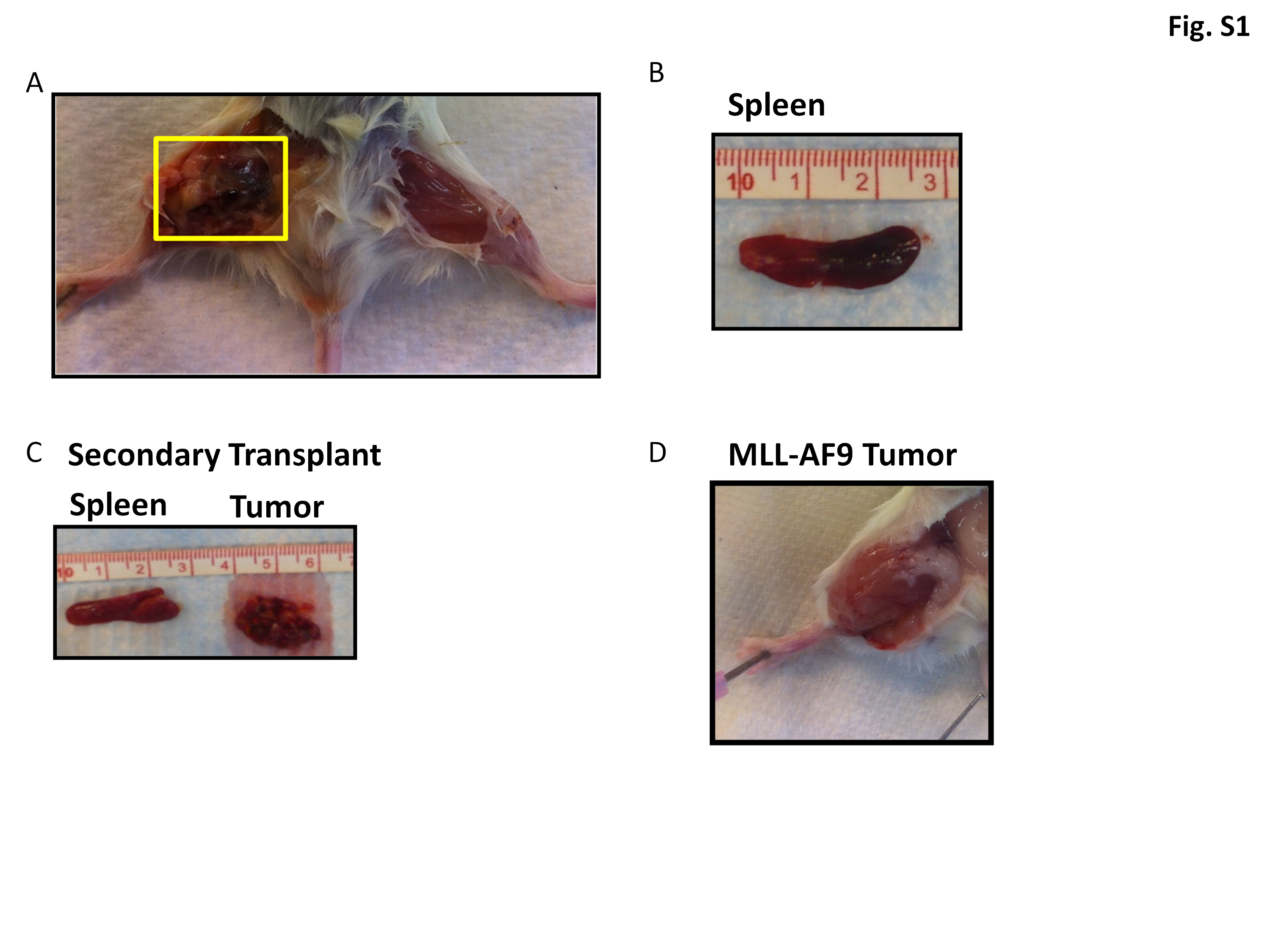

Supplement: Figure S1 — Tumorigenesis in NOD.SCID recipients of p16p19−/−; Kras(G12V) BM cells. (A) Gross anatomy of primary histiocytic sarcoma in the hind-limb of a recipient NOD.SCID mouse. (B) Enlarged spleen from a NOD.SCID mouse bearing primary histiocytic sarcoma. (C) Representative image of spleen and tumor of a NOD.SCID mouse bearing secondary histiocytic sarcoma. (D) Representative picture of NOD.SCID mice bearing MLL-AF9 leukemic BM cell- induced histiocytic sarcoma. (TIF) [file pone.0044044.s001.tif]

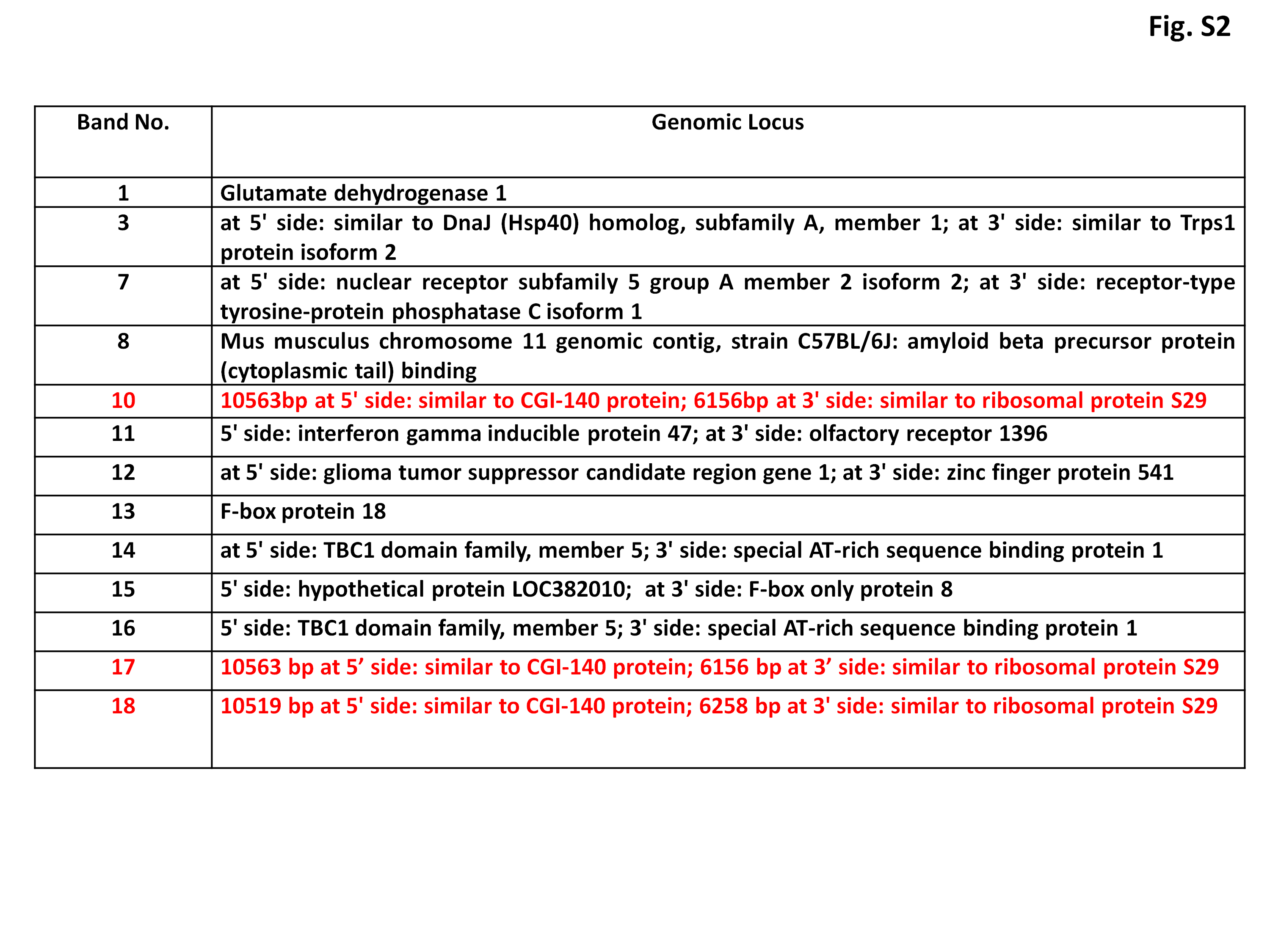

Supplement: Figure S2 — List of viral integration sites detected by LM-PCR of the histiocytic sarcoma samples from NOD.SCID mice in Fig. 6. A common integration site near the RPS29 locus is highlighted in red. Interestingly, a recent genetic screen revealed that mutation of RPS29 abolishes definitive hematopoiesis in zebrafish embryos (Burns CE et al, 2009), and loss of RPS29 affects the expression of hemoglobin suggesting a defect in red blood cells differentiation or hemoglobinization (Taylor AM et al, 2012). (TIF) [file pone.0044044.s002.tif]

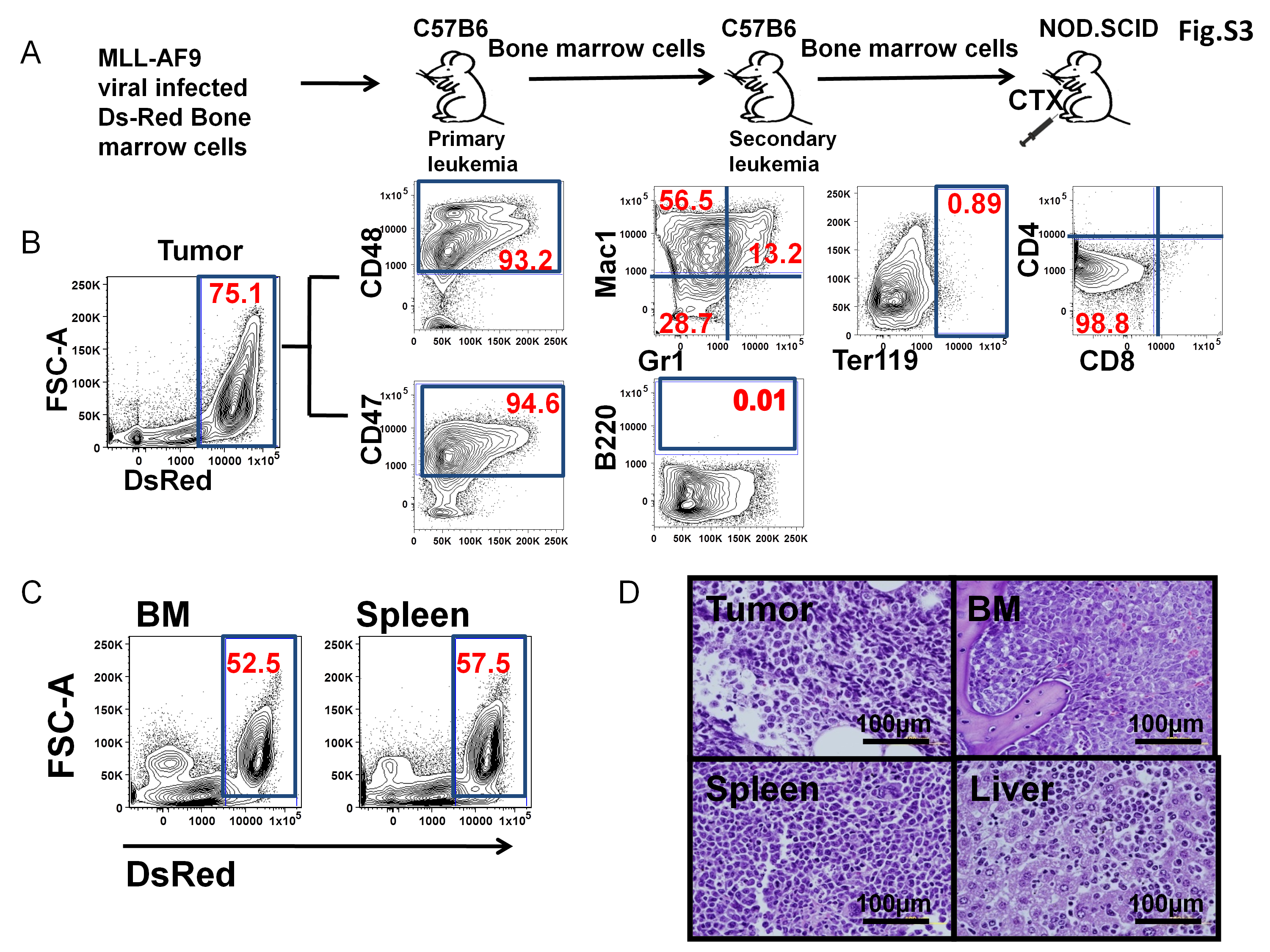

Supplement: Figure S3 — Ds-Red/MLL-AF9 leukemia cells induce histiocytic sarcoma in intramuscularly-transplanted NOD.SCID recipients. (A) Transplantation schematics for Ds-Red/MLL-AF9 transplantation. (B) Representative immunophenotypic profiling of MLL-AD9 induced histiocytic sarcomas. Plots at right are gated for live (PI-) DsRed+ cells. (C) Representative frequency of DsRed+ cells in bone marrow and spleen of mice in which histiocytic sarcomas were induced by transplantation of Ds-Red/MLL-AF9-expressing BM cells. Data collected by flow cytometry at 30 days post transplantation. (D) H & E staining of Ds-Red/MLL-AF9-induced histiocytic sarcoma, bone marrow, spleen and liver (60x). (TIF) [file pone.0044044.s003.tif]
